# Females prioritize future over current offspring in wild seasonally breeding Assamese macaques

**DOI:** 10.1098/rspb.2025.0024

**Published:** 2025-06-18

**Authors:** Shivani, Suchinda Malaivijitnond, Suthirote Meesawat, Oliver Schülke, Julia Ostner

**Affiliations:** ^1^ Department of Behavioral Ecology, University of Göttingen, Göttingen, Lower Saxony, Germany; ^2^ Social Evolution in Primates Group, German Primate Centre, Leibniz Institute for Primate Research, Göttingen, NDS 37077, Germany; ^3^ Department of Biology, Chulalongkorn University, Faculty of Science, Bangkok, Thailand; ^4^ National Primate Research Center of Thailand, Chulalongkorn University, Saraburi, Thailand; ^5^ Leibniz Science Campus Primate Cognition, German Primate Center, Leibniz Institute for Primate Research, Göttingen, NDS 37077, Germany

**Keywords:** life history, interbirth interval, trade-off, primates, maternal investment, infant survival, reproductive senescence

## Abstract

Classical work on birds by David Lack was foundational to life history theory when it uncovered a trade-off between offspring quantity and quality. Evidence for a similar trade-off was later found in singleton-bearing mammals, but its extent and underlying mechanisms are not fully understood. Here, we explore the role of adaptive reproductive scheduling and maternal energy depletion as the basis of the trade-off with data on 410 births by 104 mothers recorded over 18 years in a wild Assamese macaque (*Macaca assamensis*) population with seasonal reproduction. In any given mating season and after controlling for maternal age effects, the probability for a female to conceive was strongly predicted by the presence of a dependent offspring. The younger the current infant was, the less likely mothers were to invest in a new reproductive event, possibly to avoid stacked investment into nursing and unborn offspring. An inverse relationship between current infant survival and the conception of a new sibling points toward a shift in maternal resource allocation to future offspring. However, to avoid the energetic drain of shorter birth intervals, mothers delayed their reproductive timing within the mating season by 49 days with negative downstream effects for the next reproductive opportunity.

## 1. Introduction

Life history theory proposes trade-offs in the allocation of limited resources to different life processes such as survival, growth and reproduction [1,2]. As energy and resources devoted to one function cannot simultaneously be spent on another, individuals make crucial decisions to maximize their fitness [3]. One fundamental trade-off experienced by parents that was first described by David Lack for birds is between investing in larger numbers of offspring versus investing in the quality of each offspring [4,5].

The quantity-quality trade-off in female reproduction has been addressed in empirical studies [4,6–13], theoretical work [14,15] and a combination of both [16]. In a diverse set of reptile, amphibian, fish and mammalian taxa, increased investment in offspring number comes at the cost of offspring quality [17]. A crucial divide in how this trade-off is expressed and has been studied is between poly- and monotocous reproductive systems [18]. In polytocous species where multiple offspring are produced in a single reproductive event, clutch or litter size as quantity is negatively correlated to offspring quality measured as egg size, offspring mass, growth and survival [4,7,8,19,20]. In contrast, quantity is expressed as interbirth interval (IBI) in monotocous species that typically produce a single offspring at a time [6,10–13,21].

Primates, as a mammalian order, are particularly interesting to study life history trade-offs due to their longer lifespan and slower reproduction [22]. They invest heavily in their offspring and experience substantially longer gestation and lactation periods, resulting in extended offspring dependency on parental care compared to mammals of similar size [23–25]. This extensive parental care might come at a cost to the mother, in terms of a prolonged recovery period post-reproduction, which may delay future reproductive events [26,27]. According to the maternal depletion hypothesis, first proposed for humans [28], the number of offspring cannot easily be increased by shortening IBIs as shorter intervals between births prevent mothers from replenishing their nutritional stores adequately, which potentially impacts the quality and survival of offspring [29,30]. This claim was supported in different species of non-human primates, both in the wild [10,11,21,31,32] and in managed populations [33,34], with shorter IBIs being linked to reduced infant growth and increased infant mortality suggesting maternal depletion to mechanistically underlie the quantity–quality trade-off.

The quantity–quality trade-off is further shaped by the extent of reproductive seasonality experienced by a species or population, as a more restricted time window for successful breeding imposes stronger constraints on parental adjustments [35]. Reproductive seasonality forces a limit on how much the IBI can be extended before an entire annual season is skipped. As such, prioritizing quality over quantity by extending IBIs is less amenable in seasonal breeders than in non-seasonal breeders that can more easily fine-tune IBIs to maternal capital.

In addition, in a seasonal environment, where food resources undergo annual fluctuations, acquiring and allocating resources to meet the energetic demands of reproduction becomes more challenging [36,37]. For long-lived mammals like primates, aligning all stages of a reproductive cycle from conception through weaning within a single annual food peak is not possible [38]. Hence, females use different energy allocation and acquisition strategies to synchronize the peak in food abundance with one or another reproductive stage [32,39–41]. Income breeders use external cues like daylength to initiate ovarian cycle activity as a means to align peak lactation, i.e. the costliest phase of reproduction, with the peak in food availability so that energy income can be handed down directly to the infant [42]. Capital breeders feed their infants from stored energy that has been acquired during gestation in the food-rich season. As a function of environmental conditions, capital breeders, more than income breeders show pronounced inter-annual variation in birth rates [38]. In the income-capital continuum, some species use a mixed breeding strategy [38,43–45].

Seasonally reproducing Assamese macaques (*Macaca assamensis*) exemplify such a mixed breeding strategy and are relaxed income breeders [38]. Aspects of the capital breeding strategy are that females store energy during the food-rich season, leading to female physical condition at the onset of the mating season being correlated with their probability to conceive and fertility to vary widely from 40% to 90% of females giving birth in a year [46]. This suggests an evolved mechanism triggering conception based on physical condition. The strategy has elements of income breeding, because the peak of lactation and not gestation coincides with the most likely peak in food availability [38,46] and thus no further fat is being accumulated during gestation (unpubl. data). Females flexibly shift behavioural activity patterns depending on their reproductive phase and independent of food availability, which indicates that females balance efforts towards current needs and future ability to reproduce [47]. IBIs are bimodally distributed and cluster around one year (mean ± s.d., 13.9 ± 0.9 months) or two years (23.2 ± 0.9 months) between consecutive births [48], which further hints at a potential energetic constraint underlying female reproductive decisions. Here, we investigated predictors of conception and subsequent maternal depletion effects on offspring survival.

**Figure 1.**
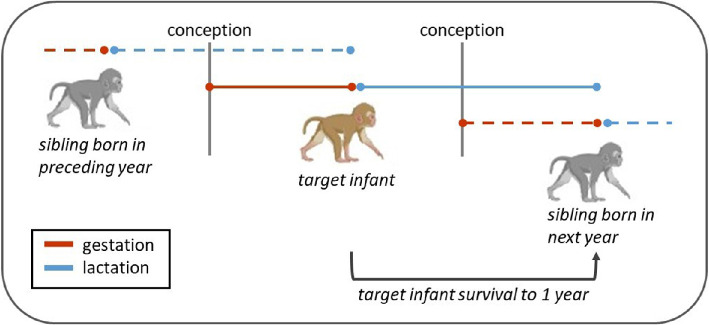
An infant’s survival (target infant in brown) may be affected both by the presence of an older or a younger sibling close in age if the IBI is close to 1 year. In case of an older sibling (born the preceding year), the mother will have to spend energy for lactation of the older sibling while investing also in the gestation of the target infant. In the case of a younger sibling (conceived the same year and born in the next year), the mother may have to direct energy resources away from the target infant’s lactation and into the gestation of the next sibling. Solid red and blue lines refer to the target infant, dashed lines refer to the respective sibling (created with BioRender).

Using long-term data from wild seasonally breeding Assamese macaques, this study examined whether females are energetically constrained to give birth every year, requiring them to conceive while still nursing their current infant (a phenomenon known as direct stacked investment [49,50]). In this population, females in good physical condition are more likely to conceive [46] and as lactation is an energetically expensive reproductive phase, mothers might not be able to replenish their nutritional stores fast enough to be able to conceive again or would have to divert energy away from lactation, jeopardizing the development of the current offspring. We predicted that (i) the probability of conceiving in a given year will be negatively associated with the presence of an offspring born during the birth season earlier in the same year. Additionally, we predicted (ii) a positive association between conception probability and the age of the previous offspring where only mothers of the oldest individuals born the same year engage in a new reproductive event. Based on the maternal depletion hypothesis [28], we expected that the presence of any close-in-age sibling competing for limited resources would be detrimental to the current infant (target infant; figure 1), so we predicted (iii) survival of a target infant until weaning at one year of age to be negatively associated with both, the birth of a sibling in the preceding year and conception of a new sibling during the target individual’s infancy (figure 1). We controlled all analyses for female reproductive senescence by including maternal age and its squared value in our models.

## 2. Methods

### (a) Study site and subjects

The study was conducted at Phu Khieo Wildlife Sanctuary (16°05−35′ N, 101°20−55′ E), which is part of a >6500 km^2^ connected network of protected forest in the northeast of Thailand and has a rich mammalian community including large herbivores and carnivores [51]. The study site ‘Huai Mai Sot Yai’ within the sanctuary is situated at an elevation of 600−800 m above sea level and comprises mostly hill evergreen forests [51]. This study used long-term demographic data collected on unprovisioned, wild Assamese macaques in their natural habitat collected over 18 years from 2006 to 2024.

Females in this population breed seasonally with a mating season from October to February and a peak birth season from April to June, where 79% of births were recorded [47]. During the study period, groups comprised a median of 46 (IQR: 39−53) individuals including several adult males, adult females and immatures. Observations began with one group in 2006, which later split into additional groups in 2012 and 2020; a second group was added in 2011, which split in 2014. At any given time, between one and five groups were followed. These groups have hugely overlapping home ranges and are surrounded by unhabituated neighbouring groups. Since 2011, observations typically involved following one group from dawn to dusk for several consecutive days before switching to the next and coming back to the same group about two to four weeks later depending on how many groups were being followed. Additional demographic data were obtained whenever a group was encountered outside its regular schedule, e.g. during intergroup encounters. In this population, all females remained in their natal groups and males dispersed at the mean age of 4 ± 1.2 years [52]. Throughout the study period, a total of 410 births from 104 adult females were recorded.

### (b) Data and analyses

Female reproductive decisions concern the onset of a new reproductive event, i.e. conception of a new infant. We retrospectively deduced conception from the observation of birth in a given season by counting back 165 days, the average gestation length reported for this population [48]. Since the species does not present overt signs of ovarian cycle activity [48], it was not possible to account for premature pregnancy terminations, which, however, should be rare based on previous endocrinological work [48].

We did not estimate the effect of very early infant death, but consider the problem to be minor. During the birth season, while following one group, we also checked the females of other groups for new infants whenever we encountered them. More importantly, between 2006 and 2011, when we followed only one group, we collected demographic information almost every day. Out of the 46 infants born during this period, only 2 (4%) died very early on days 14 and 32. We removed data on primiparous mothers (first-time mothers) for all analyses, as they did not have any previous offspring.

‘Conception’ was coded as 0 or 1 depending on whether a female gave birth or not.

‘Birth earlier the same year’ was coded as 0 and 1, depending on the absence and presence of offspring born the same year a female conceived again. If an infant died within six months from birth, we treated them as absent for the variable ‘birth earlier the same year’*,* because the mothers may have had time to replenish their energetic stores and conceive again during the upcoming mating season. Birth dates for infants were either known to the day when a female was observed one day without an infant and the next with one (21%) or were estimated as the midpoint between these events (79%). In cases where birth dates were estimated, we included the error margin that ranged from a minimum error of 1 to a maximum of 75 days in our statistical models (see below).

‘Age of the previous offspring’ was expressed in days from the day the previous offspring was born to the start of the mating season (1 October), when, based on androgen changes reported in this population [53], a female has already decided to invest in a new reproductive event even though she may conceive in her first ovarian cycle only (e.g. in January). We accounted in statistical models for uncertainty in our age estimate using the observation errors mentioned above.

‘Infant survival’ was recorded as 0 or 1 depending on whether the infant survived to its 1st birthday. ‘Birth of a sibling in the preceding year’ was recorded 1 if a sibling was born in the year before the current (target) infant was born and survived at least until six months of age. Thus, if the preceding infant died before reaching six months of age, it was coded as 0 as if no birth event had occurred earlier that year. We ran an additional model relaxing this criterion and coding any birth in the preceding year as 1 irrespective of the fate of the infant (see below and electronic supplementary material).

‘Conception of a sibling in the same year’ was determined retrospectively as 1 if a sibling was born in the birth season in which an infant reached one year of age.

‘Maternal age’ in years and its squared value were included in all models to account for possible effects of reproductive senescence, where the probability to conceive or invest enough into infant survival was low for very young mothers, high for prime-aged mothers, and declining for the oldest females.

From 410 known births, we excluded events if data were not available either on the previous or the next reproductive opportunity, because the group was not yet or not any more followed. Information on the next reproductive opportunity was also missing if the mother died within the first year of the infant’s life (seven cases). Thus, we may have very slightly skewed the sample towards higher quality, healthy females. To control for random effects of group membership, we considered six group identities.

### (c) Statistical analyses

#### (i) Bayesian modelling

We used a binomial mixed-effects logistic regression model within a Bayesian framework for our analyses [54,55]. We chose the Bayesian approach for its ability to incorporate prior knowledge into the model and better understand the uncertainty around the parameter estimates through credible intervals. All the models were fitted using the ‘brms’ package [56] in R v ‘4.3.1’ [57], which utilizes the Markov chain Monte Carlo (MCMC) sampling via ‘stan’. For all the models, we used default priors from the ‘brms’ package [58].

### (d) Model specification

#### (i) Model 1: effect of same-year birth on subsequent conception

We specified a Bernoulli distribution to account for the binary nature of our response variable, conception, which was coded as either 0 for absence or 1 for presence. To test whether a birth earlier in the same year influenced conception probability, we fitted a logistic regression with birth in the same year as fixed effect and mother identity and group identity as random effects. We included random intercepts for mother identity and group identity to account for the variability between mothers and between groups. We further included theoretically identifiable random slopes [59,60], namely birth earlier the same year (dummy coded and centred) within group identity and birth earlier the same year (dummy coded and centred) within mother identity. The specified model formula is as follows:


$$conception\;\tilde{\;}\;birth\;earlier\;the\;same\;year+mother^{\prime }s\;age+(mother^{\prime }s\;age{)}^{2}+(1+birth\;earlier\;the\;same\;year+mother^{\prime }s\;age+(mother^{\prime }s\;age{)}^{2}\;||\;GroupID)+(1+birth\;earlier\;the\;same\;year+mother^{\prime }s\;age+(mother^{\prime }s\;age{)}^{2}\;||\;MotherID)$$

The sample analyzed comprised a total of 556 reproductive opportunities, which consisted of 289 birth (when a female conceived during the mating season) and 267 non-birth events (when a female did not conceive during the mating season) from 89 different mothers. All primiparous mothers and all events without information on birth earlier in the same year were excluded.

#### (ii) Model 2: effect of age of previous offspring on conception

A continuous variable ‘previous offspring age’ was simulated to account for errors around the birthdates by drawing from a normal distribution, with distribution centred around the midpoint of the minimum and maximum age for every infant.


$$previous\;offspring\;age[i]\;\tilde{\;}\;Normal\;(mean.age[i],\;sd.age[i])$$

To ensure biologically plausible age estimates, we further bounded the values sampled from the normal distribution within the estimated minimum and maximum possible age for every infant. We standardized the simulated previous offspring age and used it in our model. We then fit a logistic regression model with standardized previous offspring age as the fixed effect and random effects for mother identity and group identity. We included random slopes for previous offspring age within mother identity and group identity to account for variation in the effect of previous offspring age on conception across mothers and groups. The specified model formula is as follows:


$$conception\;\tilde{\;}\;previous\;offspring\;age+mother^{\prime }s\;age+(mother^{\prime }s\;age{)}^{2}+(1+previous\;offspring\;age+mother^{\prime }s\;age+(mother^{\prime }s\;age{)}^{2}\;||\;GroupID)+(1+previous\;offspring\;age+mother^{\prime }s\;age+(mother^{\prime }s\;age{)}^{2}\;||\;MotherID)$$

The sample analyzed comprised a total of 533 reproductive opportunities which consisted of 281 births (when a female conceived during the mating season) and 252 non-birth events (when a female did not conceive during the mating season) from 89 mothers. We excluded 23 reproductive opportunities where females were reproductively inactive for more than three years and did not subsequently conceive. These cases were removed as extended IBIs of this length are uncommon in the study population and could disproportionately influence the model outcomes.

#### (iii) Model 3: effect of preceding and subsequent sibling on infant survival

Similar to our previous model, we specified a Bernoulli distribution for infant survival, as survival of an infant until 1 year of age was coded as 0 for death or 1 for survival. To test whether the birth of a sibling earlier in the year the current infant was conceived in and/or the conception of a new sibling during the current infant’s nursing period had an influence on the current infant’s survival (up to 1 year of age), we fitted a logistic regression model with birth of a preceding sibling (surviving at least six months) and birth of a sibling in subsequent year as fixed effects. We included mother identity and group identity as random effects with random slope of birth of a preceding sibling and birth of a sibling in subsequent year within group identity to account for the group -and individual-level variation. The specified model formula is as follows:


$$Infant\;survival\;\tilde{\;}\;birth\;of\;a\;sibling\;in\;the\;preceding\;year+conception\;of\;a\;sibling\;in\;the\;same\;year+mother^{\prime }s\;age+(mother^{\prime }s\;age{)}^{2}+(1+birth\;of\;a\;sibling\;in\;the\;preceding\;year+conception\;of\;a\;sibling\;in\;the\;same\;year\;||\;GroupID)+(1|MotherID)$$

The sample analyzed with this model consisted of 245 infants for which we had information from the previous as well as the subsequent birth seasons from 65 mothers. All infants for whom we lacked information from either the preceding or the following birth season (e.g. because a group was not under observation yet or not anymore) had to be excluded. We reran this model after relaxing the rule that previous offspring had to have survived for at least six months (see electronic supplementary material).

### (e) Model fit and diagnostics

Prior to fitting the models, we inspected the distribution of quantitative predictor variables to make sure they were symmetrically distributed. We estimated the correlations among random intercepts and slopes [60] and removed them from the model in case the absolute correlations were estimated to be one and were not identifiable (see model formula) [61].

For model fit, we used four chains and ran 2000 iterations of each chain including a warmup of 1000 iterations. We confirmed the convergence of the chains using R-hat statistics, with all values <1.01, suggesting that the chains mixed well [62]. To confirm the reliability of the estimates, we examined the effective sample size, which confirmed that the samples were effectively independent. In addition, we visually inspected the trace plot to ensure no divergent transitions and did posterior predictive checks to see if the model properly fitted the data [63]. For tables and plots, we specified the 95% credible interval given their interpretative familiarity, however, there is no need to set these as limits [64]. We generated probability of direction of estimates for the predictor variables, using the ‘bayestestR’ package [65]. These estimates are generated from the posterior distribution and range from 50% to 100% to indicate the certainty of an effect [65]. We used the ‘bayesplot’ and ‘ggplot’ package for visualization of posterior density distribution and other plots [63,66].

## 3. Results

### (a) Distribution of IBI and infant survival

One-year IBIs (57 cases, 32%) were observed less frequently than two-year IBIs (121 cases, 68%) if only those cases are considered where the first infant survived for at least six months, i.e. into the next mating season. Within the one-year IBI category, there were only three cases where a female gave birth three years in a row. Out of 245 infants, 23 (9%) died during their first year of life. Of these, 20 infants died following a one-year IBI, while the remaining three cases occurred after a two-year IBI. Among the infant deaths, 30% occurred during the first six months, while the rest (70%) occurred after the first six months. In addition, all infants that lost their mothers in their first year of life died.

### (b) Effect of same-year birth on subsequent conception (model 1)

**Figure 2.**
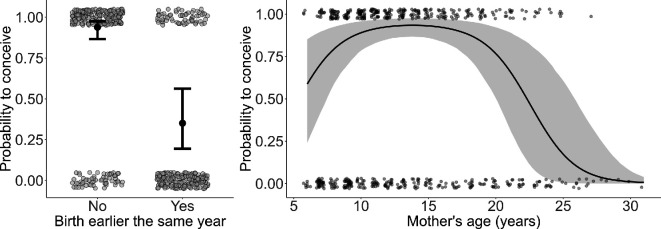
Estimated effects of birth earlier the same year and maternal age on conception probability (model 1). Left panel: estimated probability to conceive after having given birth earlier the same year or not; black dots represent the model-predicted mean infant survival probabilities with whiskers representing the 95% credible intervals; the raw data points are shown as grey dots. Right panel: maternal age effect on conception probability. The steep decline in estimated probability to conceive in a given year (black line) indicates reproductive senescence in the population. The shaded area depicts the 95% credible interval and grey dots the raw data*.*

After controlling for the effect of giving birth earlier the same year, maternal age had a non-linear effect on the probability to conceive with an estimated steep increase at young age suggesting additional trade-offs with maternal growth and a steep decrease at older ages indicative of reproductive senescence (table 1; figure 2).

**Table 1.** Results of models 1–3 investigating (1) the effect of a birth earlier the same year on conception, (2) the effect of age of previous offspring on conception, and (3) the effects of the birth of a sibling in a preceding year and the conception of a new sibling on infant survival. In all three models, maternal age and age² were included as control variables and mother identity and group identity were included as random factors. The table shows posterior estimates of population-level effects, posterior mean estimates, s.e. standard error of mean estimate, CI credible intervals, R-hat, ESS estimate of effective sample size, probability of direction of effect.

term	posterior mean estimate	s.e.	95% CI (lower-upper)	R-hat	bulk ESS	tail ESS	probability of direction of effect
***model 1: effect of same-year birth on subsequent conception***							
intercept	2.73	0.46	1.87,3.66	1.00	2066	2346	100%
birth of a sibling earlier the same year^a^	−3.34	0.64	−4.62,−2.03	1.00	2369	2408	100%
mother’s age	0.16	0.31	−0.46,0.74	1.00	2965	2102	74.6%
(mother’s age)^2^	−1.10	0.29	−1.76,−0.60	1.00	2130	1550	100%
***model 2: effect of age of previous offspring on conception***							
intercept	1.25	0.32	0.66,1.88	1.00	3627	3092	99.8%
age of previous offspring^b^	2.07	0.50	1.07,3.05	1.00	1986	2067	100%
mother’s age	0.00	0.33	−0.67,0.66	1.00	3290	2241	51.8%
(mother’s age)^2^	−1.15	0.35	−1.92,−0.55	1.00	2162	1947	99.9%
***model 3: effect of preceding and subsequent sibling on infant survival***							
intercept	4.50	0.87	2.93,6.37	1.00	1899	2240	100%
birth of a sibling in the preceding year^c^	0.37	1.24	−2.08,2.87	1.00	3277	2821	62%
conception of a new sibling^c^	−3.04	0.88	−4.81,−1.19	1.00	2432	1943	99.7%
mother’s age	−0.26	0.40	−1.14,0.46	1.00	1699	2006	74.2%
(mother’s age)^**2**^	−0.22	0.25	−0.69,0.30	1.00	2802	2829	81.6%

^a^ Dummy coded with birth earlier the same year being ‘no’ as the reference category.

^b^ *z*-transformed, mean and standard deviation of the original variable were 10.2 and 6.7 months, respectively.

^c^ Dummy coded with birth of a sibling in the preceding year and conception of a new sibling in the same year being ‘absent’ as the reference category.

The birth of a surviving offspring earlier in the same year negatively affected subsequent conception probability (table 1). If there were no offspring born earlier in the same year, the probability of a female conceiving was high, with the intercept having a posterior mean of 2.73 and the 95% CI ranging from 1.87 to 3.66 (figure 2; table 1). In contrast, the birth of an offspring in the same year was associated with a significant decrease in the odds of conception with a posterior mean of −3.34 and the 95% CI from −4.62 to −2.03 (figure 2; table 1). Back-transforming the estimates, the probability of a female conceiving when she had already given birth to a surviving offspring earlier in the same year was reduced to 35% as compared to 94% when there had not been an earlier birth in the same year, suggesting females were under energetic constraints. The probability of direction for the effect of birth earlier in the same year on conception probability was 100%, indicating that the effect is surely negative (table 1).

Conception rates varied considerably across mothers and groups. In model 1, the standard deviation of the intercept for different mothers was 0.38 (95% CI: 0.02 to 0.92) and the standard deviation of the intercept across different groups was 0.37 (95% CI: 0.01 to 1.23), which suggests other variables may be responsible for considerable parts of the observed variation.

### (c) Effect of age of previous offspring on conception (model 2)

**Figure 3.**
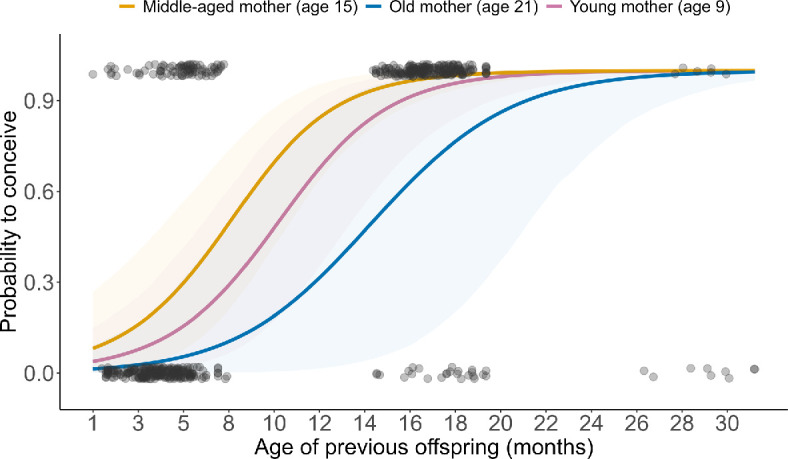
Conception probability as a function of previous offspring’s age (*n* = 533). The three solid lines represent the mean predicted probability across the posterior distribution for a female from an average group at age 9, 15 and 21 years, while the shaded regions represent the 95% credible interval around these means. Grey dots represent raw data points.

The probability to conceive increased with the continuously measured age of the previous offspring (table 1) and the estimated effect shifted with maternal age. A middle-aged female of 16 years reached a 50% probability to conceive when her previous offspring was eight months old, a young female of nine years reached that threshold only two months later and an old female of 21 years usually skipped a year when her previous offspring was less than a year old (figure 3). Notably, there are very few cases where females with 1−4 months old offspring conceived again, but a considerable number at five months and older (top left corner of the graph). This suggests that it is not the presence of a dependent offspring *per se*, but the drag it has caused on maternal energy reserves that is relevant.

**Figure 4.**
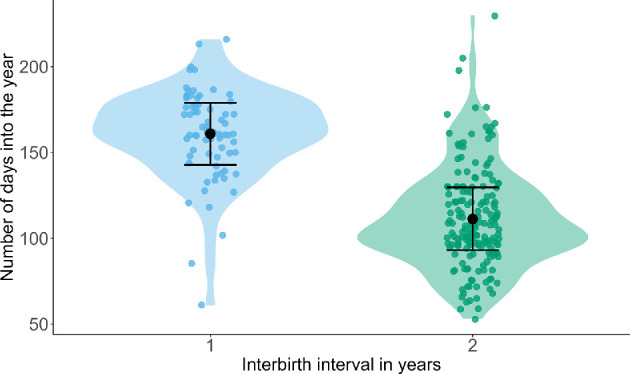
Birth date (number of days into the year) as a function of interbirth interval category (1 or 2 years; *n* = 247). The solid black dots represent the predicted mean birth date for each IBI category, with whiskers indicating the 95% credible intervals; the raw data points are shown as coloured dots*.*

We did a post hoc analysis to further investigate how the energy drain affects the timing of conception within the mating season; for birthdates after a one vs. a two-year IBI, we compared the number of days into the year (i.e. days since 1 January). For this analysis, we excluded cases with the maximum margin of error in the birthdate estimation (75 days) as this could have introduced substantial uncertainty in the timing of birth, potentially skewing the results, as well as rare cases of three-year IBIs. Following short IBIs of roughly 1 year, births occurred almost two months later in the year (mean = day 160, i.e. 8 June, 95%CI = day 143−179) compared to births following a two-year IBI (mean = day 111, i.e. 20 April, 95% CI = day 93−129; figure 4).

### (d) Effect of preceding and subsequent sibling on infant survival (model 3)

**Figure 5.**
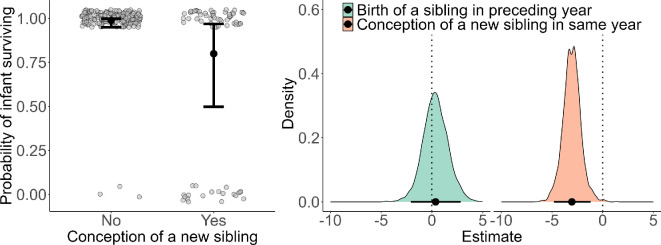
Infant survival until 1 year of age. Left panel: model-estimated infant survival probability with and without a new sibling being conceived the same year; black dots represent the model-predicted mean infant survival probabilities, with whiskers representing the 95% credible intervals; the raw data points are shown as grey dots. Right panel: density plot of the posterior distribution of model parameters for infant survival (*n* = 245) on a log-odds scale; the horizontal black line represents the 95% credible interval and black dots on the line indicate the posterior mean. The vertical dashed line at 0 represents the null value and the estimates to the left indicate a negative association and to the right a positive association*.*

Model 3 did not generate support for maternal age, its squared value, or the presence of a preceding sibling (that had survived for at least six months) affecting the current infant’s survival (high uncertainty of effect in all cases; figure 5; table 1). In contrast, the conception of a sibling during the target infant’s first year of life, and thus during this infant’s nursing period, was associated with a significant decrease in the odds of survival (posterior mean = −3.04, 95% credible intervals = −4.81 to −1.19) with a 99.7% probability of effect direction (table 1; figure 5). When a mother conceived during her lactation period, the infant’s survival probability was reduced by 19% to 0.80 from 0.99 when there was no conception (back-transformed estimates; figure 5).

We reran the model relaxing the criterion of survival of the preceding sibling of at least six months (electronic supplementary material, table S1 and figure, S1). As in the original model, the conception of a subsequent sibling had a strong negative effect on the survival of the target infant. In addition, there is weak support for a negative effect of presence of a preceding sibling (posterior mean = −0.94, 95% credible interval = −3.07 to 1.07, 84% probability for a negative effect).

We also found that the baseline infant survival probability varied across mothers and groups, with pronounced variation across mothers (s.d. intercept 1.05, 95% CI: 0.07, 2.54) and across groups (s.d. intercept 0.65, 95% CI: 0.01, 2.34), which suggest additional variables affecting the response.

## 4. Discussion

Despite the caveat that considerable amounts of variation were left unexplained, our analyses strongly suggest that energetic constraints shaped female reproductive decisions in this population of seasonally reproducing Assamese macaques. The presence and age of an offspring strongly predicted a mother’s probability to conceive again, with too little time to replenish energy reserves precluding investment in the next offspring. When mothers conceived again early (i.e. in the next breeding season), this came at a cost to offspring quality, as survival of the current offspring was reduced, suggesting maternal depletion underlying the quantity-quality trade-off. This result is consistent with findings in other seasonally breeding wild and free-ranging populations of singleton-bearing primates, where increasing offspring quantity by reducing IBIs is associated with reduced offspring quality, evident from higher infant mortality [21,34].

Infant survival was associated with the presence of a sibling mainly in one direction, i.e. survival decreased when a new sibling was conceived while the current infant was still being nursed. This overlap of lactation and early gestation may present a significant energetic burden to females. While gestation requires energetic investment to build and maintain tissues to support the growing foetus [67,68], during lactation energy is needed to meet the nutritional demands of the current infant [68–70]. In contrast, the occurrence of a sibling birth event preceding the current infant had a much weaker and less certain association with infant survival, and only appeared in the modified model including preceding siblings that died very early (i.e. prior to the conception of the target infants), which is possibly an indicator of poor maternal condition through both reproductive events.

Assamese macaque females, as relaxed income breeders [38], use the pre-mating season to conserve energy by increased resting [47] and accumulate fat stores in preparation for the energetic demands of reproduction [46]. There was a strong effect of the current infant on the probability to conceive and this effect was stronger the younger and hence the more demanding the current offspring was. Thus, in the first few months after parturition, mothers likely experienced lactational amenorrhoea, a physiological inability to resume ovarian cycling [71,72]. However, after this period, females may have had a choice to engage in a new reproductive event or not, for which we assume an evolved mechanism that triggers conception based on maternal condition [37,38]. If a lactating female conceived, she seemed to withdraw energy resources from the current infant, perhaps by providing milk at lower quantity or quality [21,73]. These mothers did not compromise the new reproductive event and seemed to allocate sufficient energy towards gestation, ensuring that the younger sibling did not suffer reduced survival from having a close-in-age older sibling. Similar patterns have been found in wild chimpanzees, where females sometimes wean their offspring early to begin investing in the next [10], and in free-ranging rhesus macaques, where a younger sibling may take away maternal resources from older offspring, resulting in increased mortality risk [34,74]. It is difficult to quantify the energy flow from the mother to a nursing infant [21,75], but these mothers seem to avoid heavily stacking investment into lactation and gestation as described (e.g. in colobus monkeys in response to instability in multimale groups [49]).

The effect size of the quantity–quality trade-off may seem small, with a 19% reduction in infant survival for adding another offspring, which seems to contradict the idea that seasonal breeders face stronger constraints. However, the full extent of consecutive reproductive timing has at least three additional elements, including forgone opportunities and consequences for reproductive tenure of mother and offspring. First, whether a female conceives again in the same year she already gave birth depends on timing within the season. Only if she gave birth early in the season, the current infant will be older than 4−5 months and she may conceive again. All females that gave birth late in the season in July and August, did not even get close to weighting quantity against quality—they did not conceive. Should a mother of a 4−5-month-old infant conceive, she will do so late in the mating season to maximize investment in current offspring before diverting energy towards a new gestation event [48]. Therefore, the subsequent offspring will be born so late in the next birth season that the mother cannot conceive that year again and has to forgo an opportunity. That females sometimes skip a year may be thought of as a consequence of the quantity-quality trade-off, where survival chances of the current offspring would be so low, that a mechanism evolved that makes the mother pre-emptively forego conception. Without this condition-dependent trigger, which is absent in strict-income breeders, shorter IBIs would be more frequent, and infant mortality would increase. We propose this evolved mechanism because at the onset of the mating season, towards the end of the rich season, females are at their peak physical condition, and even lactating females are not starved to a degree that prohibits ovarian cycling.

The second crucial part missing in the picture is are consequences of reproductive scheduling for maternal survival in the sense that maternal depletion from short IBIs may jeopardize her longevity [76,77]. For the study population, data have not yet been accumulated for such analyses. A regression of number of infants surviving over number of years a mother was observed, produced a linear best fit that suggests females reproducing faster are not dropping out of the sample earlier and thus not paying a lifetime cost of fast reproduction (electronic supplementary material, figure S2). On the contrary, the residuals of females that had already died (*n* = 26) were smaller (mean ± s.d. = −0.68 ± 1.16) than those of females still living (*n* = 61; mean ± s.d. = 0.29 ± 0.76), which may be a consequence of reproductive senescence.

**Figure 6.**
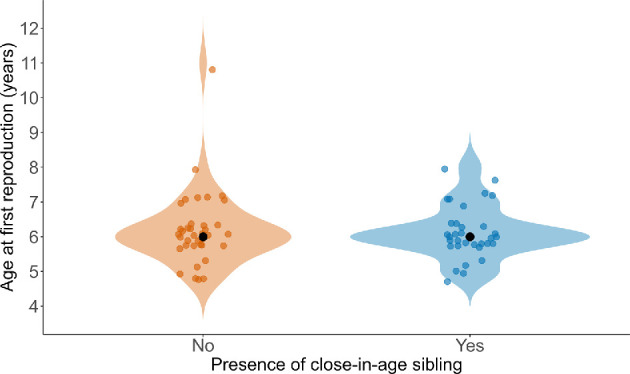
Female age at first reproduction did not differ between females that did or did not have a close-in-age sibling (*n* = 70 females that were born during the study and survived to their first reproductive event)*.*

Third, for the offspring we have explored only the immediate costs in terms of survival to one year. Having a close-in-age sibling is associated with shorter life span in yellow baboons [78,79] but with longer life spans in olive baboons [80]. Again, our study is too young to explore effects on offspring longevity for the vast majority of individuals included here. More data were available for offspring age at first birth, which is a strong predictor of reproductive life span [81–83]. Age at first birth did not differ between females that had a close-in-age sibling or not (figure 6). A flexible strategy yielding a mix of 1 year and 2 year IBIs [48] sensitive to maternal energy depletion [21,84] may be particularly effective in habitats with only moderately predictive food resource abundance such as the study area in Thailand [46].

This study was based on 18 years of long-term demographic data available for a wild Assamese macaque population. The analyses could be enriched by additional data on maternal condition and environmental factors [85,86], but these were available only for different smaller parts of the study period. Access to food affects energy intake and glucocorticoid levels in this population [46,87], which may generate variation in physical condition that explains a part of the variation in conceptions and infant survival. Demonstrating condition-dependent conception among females that gave birth earlier the same year, would provide further support for an adaptive mechanism timing consecutive reproductive events. If condition cannot be directly measured, one may predict it from a complex interaction term, where at low (and less so at high) food availability in the environment, individuals in larger groups will suffer more due to scramble competition [88], which will be exacerbated for lower-ranking females [89] through contest competition, particularly if they lack strong, tolerant and cooperative relationships that could provide them with a buffer against competitive exclusion [90]. Furthermore, developmental effects may affect the ability of females to invest in a new reproductive event and to see it through until successful weaning [91,92]. The age effects identified here suggest that additional life-history trade-offs may be relevant. Conception probability was low for the youngest mothers and increased over the first three years of female reproductive careers, which suggests females balancing somatic growth against reproduction as seen in other macaques that continue to gain muscle and body mass after their first birth [73,93]. Reproductive senescence may also have affected our results [94,95], but the degree to which the observed pattern is driven by a few females that had already been multiparous at the onset of the study will have to be explored. The causal relationships between these variables are complex and require explicit modelling before more holistic analyses can be designed, but most effects are mediated via maternal ability to invest energy into reproduction. As such, we do not expect results of this study to drastically change with inclusion of additional variables, but to provide more direct tests of the underlying mechanisms.

## Data Availability

The data and code are available at the Dryad Digital Repository [96]. Supplementary material is available online [97].
